# META analysis on the effect of taijiquan on improving negative psychological symptoms of college students and the optimal dose

**DOI:** 10.3389/fpubh.2022.1032266

**Published:** 2022-12-16

**Authors:** Zhihao Du, Xiuli Zhang, Hao Qin, Ruiqi Wang, Yunxia Bai, Xiaonan Yao

**Affiliations:** ^1^School of Physical Education and Exercise, Beijing Normal University, Beijing, China; ^2^School of Physical Education (School Headquarters), Sports and Medical Integration Development Research Center, Zhengzhou University, Zhengzhou, China; ^3^School of Computer Science, Baotou Medical College, Baotou, China; ^4^School of Sociology, Sanya University, Sanya, China

**Keywords:** Tai chi, negative psychology, depression, anxiety, college students, meta-analysis

## Abstract

**Background:**

Taijiquan, as a physical and mental exercise, can improve the negative psychology of college students. However, it is still controversial, and the optimal exercise dose of taijiquan to interfere with negative psychology has not been evaluated.

**Objective:**

This study is aimed at systematically evaluating the effect of taijiquan therapy on improving negative psychological symptoms of college students and its optimal intervention dose.

**Methods:**

Search databases such as Web of Science, Embase, PubMed, CNKI, WFSD, etc. Collect high-quality relevant RCT studies. After screening, extracting, coding and counting the data, a META analysis is done through Review Manage 5.3 and Stata 15.0 software. PICOS established the eligibility criteria to select the studies as follows: (i) population - non-clinical of college students; (ii) intervention - taijiquan intervention; (iii) comparison - taijiquan intervention group and regular physical activity group; (iv) outcomes - depression, anxiety; and (v) study design - randomized controlled trial.

**Results:**

A total of 12 articles and 1,000 samples were included. All of the participants are college students. Taijiquan therapy can significantly reduce the depression and anxiety symptoms of college students [SMD = −0.53, 95% CI (−0.82, −0.23)], [SMD = −0.49, 95% CI (−0.90, −0.09)], with statistical significance (*P* < 0.05). Subgroup analysis shows that: there is a precise focus on depression and anxiety symptoms. The intervention period is more than 12 weeks, and the best effect appears when people practice 3 times a week. The best single intervention time for depression symptoms is 60 min, and for anxiety symptoms 80–90 min. It is found that taijiquan combined with mindfulness intervention can significantly reduce negative psychological symptoms like depression and anxiety of college students than single taijiquan intervention. Funnel plot combined with sensitivity analysis, Begg, Egger test showed no publication bias.

**Conclusion:**

Taijiquan intervention can effectively improve the negative psychological symptoms of college students, and it has great promotion value in colleges and universities.

**Systematic review registration:**

https://www.crd.york.ac.uk/PROSPERO/, identifier: CRD42022314071.

## Introduction

The 2020 Blue Book of Chinese National Mental Health shows that the detection rate of depression among adolescents is 24.6%, of which severe depression is 7.4%. The detection rate of depression among college students is higher than that of the other population. The proportion of psychological disorders among college students around the world is high and there is an increasing trend year by year (1). Most adults who meet the criteria for Major Depressive Disorder (MDD) have experienced relative symptoms in childhood, adolescence, or early adulthood. Even mild depressive symptoms in adolescence are associated with an increased risk of major depression and suicide later in life. Mental health affects not only current studies, but also work and mental health in adulthood (2). In 2021, the WHO issued the Guidelines for the Promotion and Prevention of Adolescent Mental Health. It can be seen that the psychological problems Chekroud of college students are now extremely serious and the prevention and rehabilitation of early negative psychological symptoms of college students is extremely important.

The research on the use of exercise intervention to treat mental illness is increasing nowadays. Scholars such as Raglin (3), Chekroud et al. (4), and De Sousa et al. (5) believe that regular physical activity can reduce depression, anxiety, and reduce the risk of suffering from mental illness. Among them, taijiquan therapy is gradually accepted internationally and will be included in the list of World Intangible Cultural Heritage in 2020 (6). It takes taijiquan and yin-yang dialectical thinking of Confucianism and Taoist philosophy in traditional culture as the core concept. And it has the function of cultivating temperament and strengthening the body. The effect of promoting physical and mental health is remarkable. Hu et al. (7), Guo et al. (8), and many other scholars declare the positive significance of taijiquan on mental health.

So far, in the aspect of systematic review, domestic researcher has conducted a meta-analysis on the improvement of mental health by taijiquan (9). The research objects include multiple groups such as college students, middle-aged women, elderly women, patients with irritable bowel syndrome and residents of earthquake-stricken areas. But there is no following study of optimal training practices. The systematic review of Wang et al. is based on studies published in English without meta-analysis (10). It is reported that taijiquan' s psychological effects are not sure. Another review by this author reports that taijiquan appears to be associated with improving mental health, but cautioned that the inclusion of studies with different designs, different outcomes, and insufficient controls, the low quality of included studies compromised the reliability of the results (11).

For the prevention and control of negative psychology of college students, it is obviously unrealistic to apply psychotherapy to college students on a large scale. Compared with the toxic and side effects of drug treatment, the effect of physical exercise on improving the negative psychology of college students has been paid more and more attention. In addition to aerobic exercise can effectively improve the negative psychological state of college students, this study further verified the effect of taijiquan on negative psychology, and the best exercise dose for taijiquan to affect negative psychology of college students. In addition, while strictly screening the studies, this study strictly followed the PRISMA requirement process to report (12), and then adjusted the variable analysis to find out the optimal exercise dose, which provided higher methodological quality for taijiquan in the treatment of negative psychological disorders of college students and more rigorous evidence-based support. In addition, in this study, while strictly screening the studies, the report is conducted in strict accordance with the procedure required by PRISMA (12). The adjustment variables are employed to find out the optimal exercise dose. It provides a higher methodological quality and a more precious support for taijiquan in the treatment of negative psychological disorders of college students.

## Research objects and methods

This study applied for and has been registered on the PROSPERO platform with the registration ID of CRD42022314071.

### Inclusion criteria

The following are the exclusion criteria of the literature: (1) non-experimental literature; (2) duplicate publications; (3) literature in which experimental outcome data were not presented in (x+s) form; (4) subjects were other than college students not in medical clinical status: (5) incomplete data descriptions or untransformable.

In addition, the PICOS method is used to select relevant studies (Table 1). The selected research must be published in an academic journal in English or Chinese. Studies are considered eligible if the results are discussed in terms of negative psychology among college students.

**Table 1:** PICOS-based eligibility criteria (participation, intervention, comparison, outcomes, and study design).

**PICOS**	**Criteria**
Participation	Non-clinical of college students
Intervention	Taijiquan intervention
Comparison	Taijiquan intervention group and regular physical activity group
Outcome	Depression, anxiety
Study design	Randomized controlled trial

### Literature search

Retrieval is based on the PICOS principle of evidence-based medicine, and searches in databases such as Embase, PubMed, Web of Science, CNKI, WFSD, etc. The subject headings are searched for free words through PubMed, combined with the search, and the keywords include: (“TaiChi”OR“Taijiquan”) and (“University”OR“undergraduate”OR “College student” OR“Youth”) and (“Psychological”OR“mental”OR“Depression” OR“Anxiety”OR“intervention”OR“intervention trial”) and other Boolean logic search. The retrieval time was from the time when the database was established to January 2022. At the same time, relevant information was searched through the included references, and the unpublished academic literature was not retrieved. Take PubMed as an example, (see Box 1).

**Box 1:** PubMed retrieval strategy.

#1 (“negative”(All Fields) OR “negatively”(All Fields) OR “negatives”(All Fields) OR “negativities” (All Fields) OR “negativity”(All Fields)) AND “psychology”(MeSH Terms) #2 “Psychology”(Title/Abstract) OR “Depression”(Title/Abstract) OR “Anxiety”(Title/Abstract) OR “negative emotion”(Title/Abstract) OR “negative psychology”(Title/Abstract) #3 #1 OR #2 #4 “Psychology”(Title/Abstract) OR “Depression”(Title/Abstract) OR “Anxiety”(Title/Abstract) OR “negative emotion”(Title/Abstract) OR “negative psychology”(Title/Abstract) #5 “College”(Title/Abstract) OR “Student”(Title/Abstract) OR “University”(Title/Abstract) #6 “bodyexercise”(Title/Abstract) OR “Taichi”(Title/Abstract) OR “Qigong”(Title/Abstract) OR “Taijiquan”(Title/Abstract) #7 “randomized controlled trial” (Publication Type) OR “controlled clinical trial” (Publication Type) #8 #3 AND #4 AND #5 AND #6 AND #7

### Literature screening process

The collected literature is imported into Endnote X9.1 software according to the research strategy. Duplicate bibliographies in the database are excluded, and then two researchers screen according to the title and abstract. Further screening is carried out through detailed reading of the full text according to the established inclusion and exclusion criteria. Two researchers cross-check their respective screening results. If they are consistent, the bibliographies are included in the study. If the checks are inconsistent, a third researcher enters into to negotiate until they reach a consensus. Information is extracted from the final included literature, using a pre-determined method and recording the literature risk of bias.

### Data extraction

Mainly include: (1) Basic information of the included literature (title, first author, publication year, and journal); (2) Subject characteristics (sample size, age, gender, race); (3) Exercise interventions (exercise type, intensity, duration, frequency, time of intervention); (4) Intervention measures in the control group; (5) Relevant information on literature quality evaluation; (6) Outcome indicators and main findings.

### Evaluation of risk of bias in the included literature

Following the Cochrane Collaboration guidelines (13), the instrument of recommended in the handbook of Cochrane 5.1 is applied and two researchers independently assessed the quality of included literature. The evaluation consists of seven levels. 1. Generation of random sequences; 2. Allocation concealment; 3. Blinding of subjects and experimenters; 4. Blinding of outcome assessors; 5. Integrity of the resulting data; 6. Selective reporting; 7. Other source of risk. It assessed each study on three dimensions of high risk of bias, unknown risk of bias and low risk of bias. If the assessment results are inconsistent, discuss with the third researcher to resolve.

### Statistical analysis

The outcome indicators in included literature are continuous variables, and different tools are used for the same indicator. So they are expressed as standardized mean differences (SMD). The baseline is uniformly adjusted to α = 0.05. The random-effects model was used to carry out the meta-analysis (14). The I^2^ statistic was employed to assess the heterogeneity. *I*^2^ values were considered having low (or < 50%), moderate (or 50–75%), or high heterogeneity (or >75%) (15). If the heterogeneity is large, a sensitivity analysis should be performed first, and the sources of heterogeneity should be examined by excluding the literatures one by one to observe whether the results have changed significantly. The analysis compares intervention characteristics as sub-points and observes the results. Egger's test (16) and Begg's test (17) determine whether there is a risk of bias. The threshold for statistical significance was defined as *p* < 0.05. All analyses used comprehensive meta-analysis software (Biostat Inc., Englewood, NJ, USA; version 2).

## Findings

### Literature search and screening

**Figure 1:**
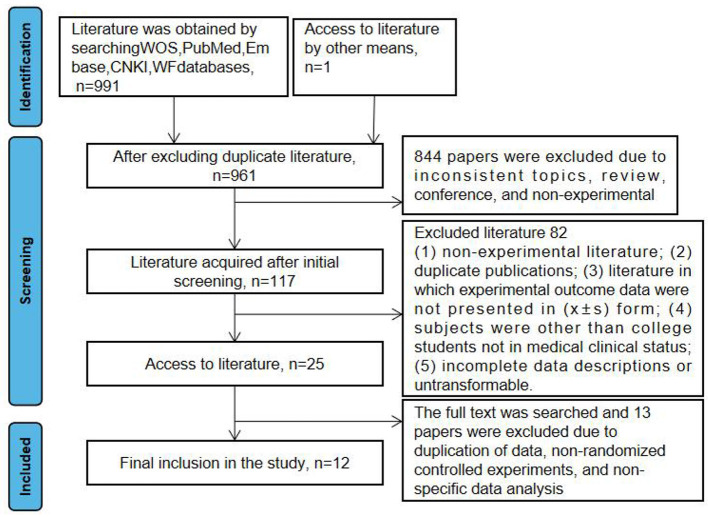
PRISMA selection process summary.

A total of 991 literatures were obtained from the preliminary screening, and were excluded one by one through full text search, reading, and quality evaluation. Finally, 12 studies were included. The screening process and screening results are shown in Figure 1.

### Basic characteristics and quality evaluation of the included literature

According to the inclusion criteria, 12 articles were selected, including 10 articles on depression and seven articles on anxiety. The number of subjects for depression and anxiety was, respectively, 890 and 673. The basic information of the first work, sample size is shown in Table 2 for details.

**Table 2:** Includes the basic information of the literature.

**References**	**Sample size (T/C)**	**Intervene type**	**Nation**	**Gender (M/F)**	**Duration, frequency of intervention**	**Measuring tools**	**Index**	**Focus of intervention**
Li (18)	127/118	Japanese	China	NR	20 weeks, 1–5 times/week, 50 min/time	SCL-90	,	NR
Yang (19)	51/51	24-type	China	NR	2 months, 3 times/week, 60 min/time	SCL-90	,	NR
Mao (20)	52/52	24-type	China	0/104	18 weeks, 3 times/week, 60 min/time	SDS, SAS	,	Enter the mental state of “relaxing and falling asleep” and “replacing a hundred thoughts with one thought”
Li and Yin (21)	20/18	24-type	China	0/38	8 weeks, 3 times/week, 60 min/time	POMS		Motivators (perceptions of self-ability, beliefs, attitude building, etc.)
Chen et al. (22)	18/18	24-type	China	0/36	16 weeks, 3-4 times/week, 60 min/time	CES-D		NR
Du et al. (23)	16/16	24-type	China	16/16	2 months, 2 times/week, 60 min/time	SCL-90	,	“Healthy Model of Body, Mind and Whole Person”, (the content of intervention exercises in all aspects of body, emotion, and thoughts.)
Su (24)	40/10	24-type	China	25/25	12 weeks, 2 times/week group + 5 times/week group, 60 min/time	BDI		NR
Wang (25)	23/18	24-type	China	NR	12 weeks, 3 times/week, 60 min/time	SCL-90		NR
Hua and Sun (26)	67/20	24-type + Mindfulness	China	1/66	12 weeks, once/week, 80 min/time	DASS	,	Emphasis on the concept of mindfulness (understanding the meaning of “intelligence, qi, form, spirit”, etc.)
Hu (27)	30/30	24-type	China	NR	18 weeks, 4 times/week, 65 min/time	SAS		Abdominal breathing + music + encouragement in many ways
Park and Kim (28)	24/26	Taijiquan+ Mindfulness	South Korea	4/46	7 weeks, 3 times/week, 60 min/time	S-AI		Meditation and breathing
Zhang et al. (29)	32/32	Taijiquan + Mindfulness	China	23/41	8 weeks, 2 times/week, 90 min/time	PHQ-9		NR

, Depression; , Anxiety; NR, Not reported; SCL-90, symptom checklist 90; SAS, self-rating anxiety scale; SDS, self-rating depression scale; DASS, depression, anxiety, and stress scale; PHQ-9, patient health questionnaire-9; BDI, Beck depression inventory; HAMD, Hamilton depression scale; POMS, profile mood states; CES-D, self-report depression Scale; S-AI, state trait anxiety inventory.

### Quality assessment and risk of bias

Methodological quality was examined with the quantitative assessment instrument referred to as “QualSyst” (30), which comprises a total of 14 items (see Table 3). The scoring procedure depends on the extent to which a particular criterion is satisfied (no is 0, partial is 1, yes is 2). The final summary value of every study was computed. The calculation was carried out by two independent reviewers. A third senior reviewer was also requested to give an opinion for obtaining a reasonable consensus. Scores of ≤ 55, 55–75, and ≥75% indicated low, medium, and high quality, respectively.

**Table 3:** “Qualsyst” of quality assessment.

**Ji et al. (18)**	**Yang et al. (19)**	**Mao et al. (20)**	**Li and Yin (21)**	**Chen et al. (22)**	**Du et al. (23)**	**Su (24)**	**Wang (25)**	**Hua and Sun (26)**	**Hu (27)**	**Park and Kim (28)**	**Zhang et al. (29)**	**Publication**
2	2	2	1	1	2	1	1	2	1	1	1	Question/ objective described
2	2	2	2	2	2	1	2	2	2	2	2	Appropriate study design
1	2	1	1	1	2	1	1	2	1	2	2	Characteristic sufficiently described
1	1	1	NA	1	1	1	NA	1	1	2	2	Random allocation
0	0	0	0	0	0	0	0	0	0	1	1	Researchers blinded
0	0	0	0	0	0	0	0	0	0	1	1	Subjects blinded
1	1	1	1	2	2	2	1	2	1	2	2	Outcome measures well-defined and robust to bias
1	1	1	2	2	2	1	2	1	2	2	2	Appropriate sample size
2	2	2	2	2	2	2	2	2	2	2	2	Analytic methods well-described
1	2	1	1	2	2	1	1	2	1	2	2	Estimate of variance reported
0	0	0	0	1	1	0	0	1	0	1	1	Controlled for confounding
2	2	1	1	2	1	1	2	2	1	2	2	Results reported in detail
2	2	1	1	2	1	1	2	2	1	2	2	Conclusion supported by results?
Medium	Medium	Medium	Medium	Medium	High	Medium	Medium	High	Medium	High	High	Rating

**Figure 2:**
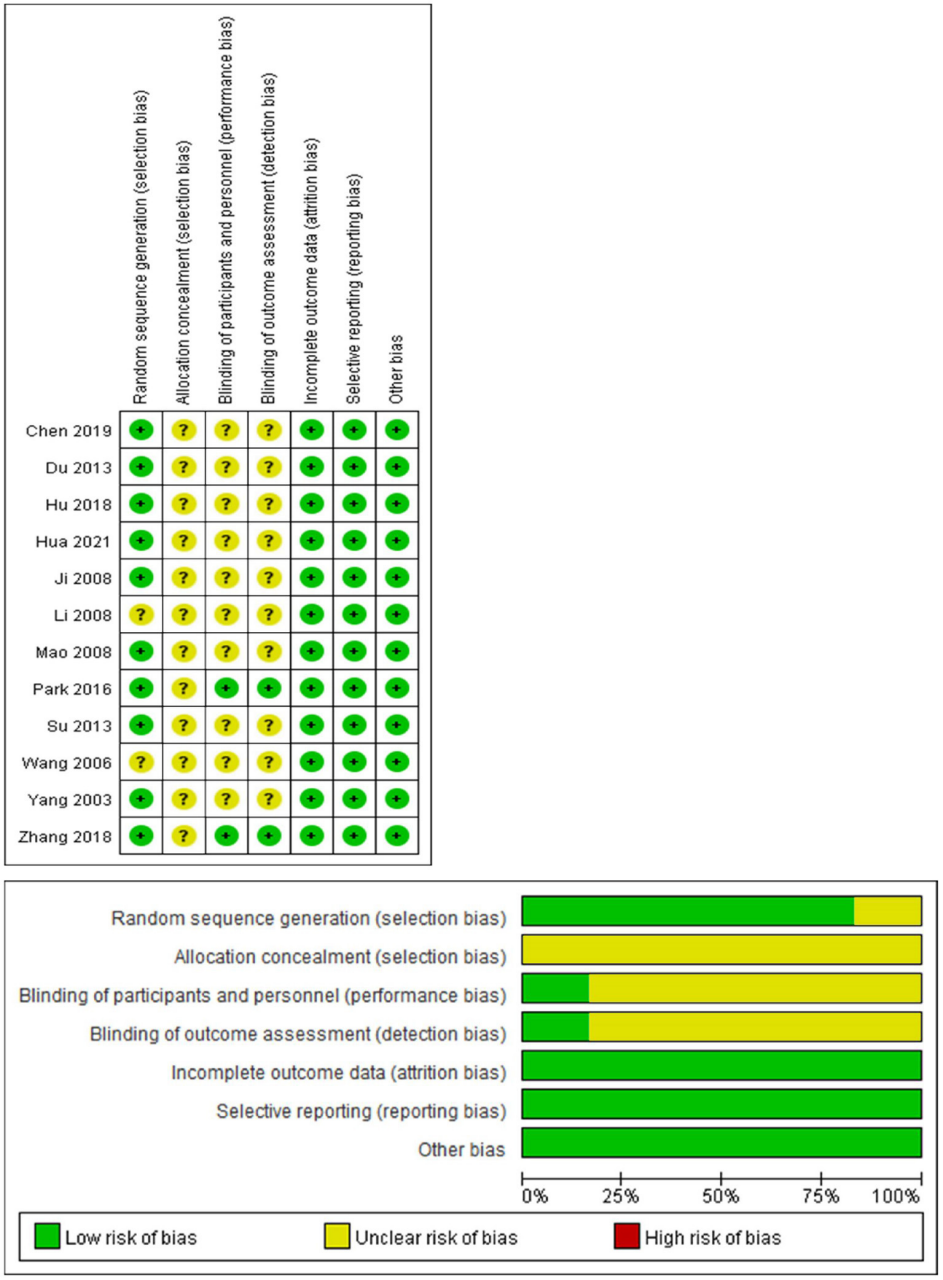
Risk of bias assessment using Rob 2 for included studies.

Of the included literature, two articles (28, 29) described specific random sequence methods and nine articles (18–27) mentioned only the word “random”; two articles (28, 29) described allocation scheme concealment and the rest did not mention it; the outcome data of the 12 studies were complete, not selectively reported, and not otherwise biased Source. Most experiments were not blinded due to the specificity of taijiquan training. The results of the quality evaluation of the literature (see Figure 2).

### META result analysis

#### Depression indicators

**Figure 3:**
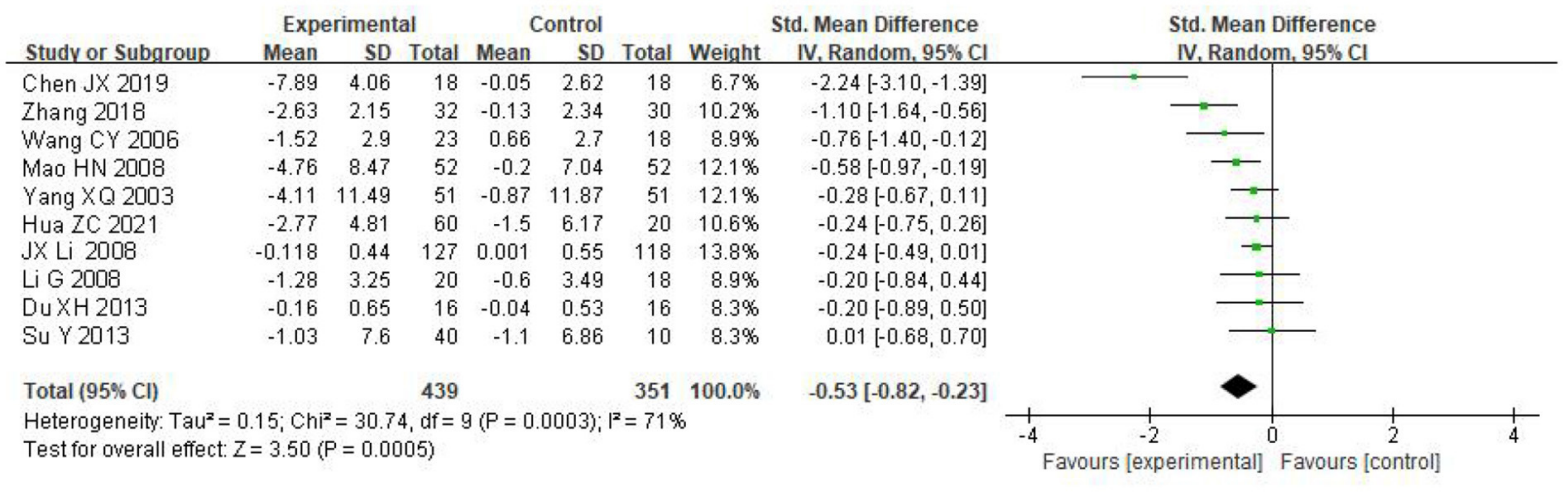
Forest plot of the effect of taijiquan on college students' depression.

Ten articles included, and a total of 890 subjects were included with a total sample size of 439 cases in the experimental group and 351 cases in the control group. The heterogeneity test showed that the heterogeneity was large (*I*^2^ = 71%, *P* = 0.0005), and the effect values were combined using a random effect model. The results showed that the depression score of the taijiquan group was significantly lower than that of the control group [SMD = −0.53, 95% CI (−0.82, −0.23), *P* = 0.0005], indicating that taijiquan intervention training has a significant effect on reducing the depression level of college students (see Figure 3).

#### Anxiety indicators

**Figure 4:**
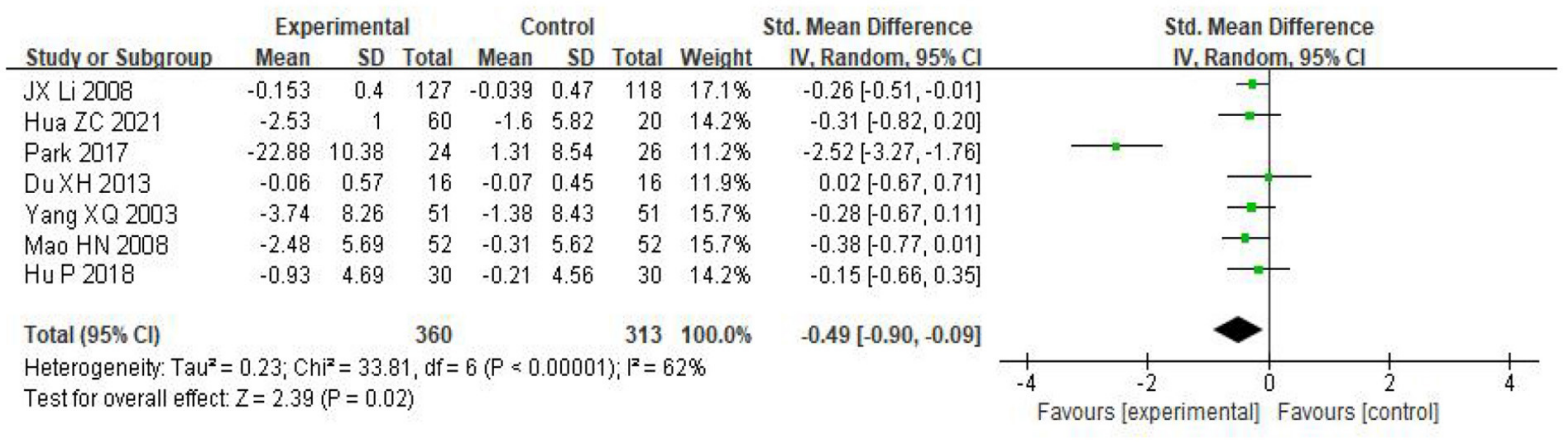
Forest plot of the effect of taijiquan on college students' anxiety.

Seven articles were included, with a total of 673 experimental subjects with 360 in the experimental group and 313 in the control group. The heterogeneity test showed that the heterogeneity was large (*I*^2^ = 62%, *P* < 0.00001), and a random effect model was used to combine the effect values. The results showed that the anxiety score of the taijiquan group was significantly lower than that of the control group [SMD = −0.49, 95% CI (−0.90, −0.09), *P* = 0], indicating that taijiquan intervention training has a significant effect on reducing the anxiety level of college students (see Figure 4).

### Subgroup analysis

#### Intervention period

Among the 10 studies included in depression indicators, the combined effect of seven studies with an intervention period of ≤ 12 weeks was [SMD = −41, 95% CI: (−0.62 to −0.21), *P* = 0]. For three studies exceeded 12, the combined value of the study effect of the intervention cycle of the period was [SMD = −0.45, 95% CI (−0.66, −0.25), *P* = 0]. Compared with the control group, the effects of taijiquan in the two intervention cycles on depression had a significant statistical difference, and there was a statistical difference between the groups (*P* < 0.05), that is, the intervention period of more than 12 weeks could significantly reduce the depression level of college students.

Among the seven studies included in the anxiety index, the combined effect value of 4 intervention period studies with a duration of ≤ 12 was [SMD = −0.48, 95% CI: (−0.75, −0.22), *P* = 0.174]. For three studies >12 weeks, the combined value of the intervention cycle study effect was [SMD = −0.28, 95% CI: (−0.47, −0.08), *P* < 0.001]. Compared with the control group, the intervention cycle of ≤ 12 weeks taijiquan had a difference in the effect of anxiety. There was no statistical significance (*P* > 0.05), and there was a statistical difference between the groups (*P* < 0.05), that is, the intervention period of more than 12 weeks could significantly reduce the depression level of college students.

#### Training frequency

Of the 10 studies included in the depression index, three had a combined effect of 1–2 weekly training frequency [SMD = −0.55, 95% CI: (−0.88, −0.23), *P* = 0.001]. The combined effect of training frequency 3 times per week was [SMD = −0.58, 95% CI (−0.81, −0.34), *P* = 0]. Compared with the control group, the effect of training frequency on depression was significant statistical difference. And the comparison between groups was statistically significant (*P* < 0.05), suggesting that the improvement of depression was ranked as 3 times > 1–2 times for different training frequencies.

Among the seven studies included in the anxiety index, the combined effect of two studies with training frequency 1–2 times per week was [SMD = −0.1, 95% CI: (−0.51, −0.31), *P* = 0.55]. The combined value of the study effect of training frequency 3 times per week was [SMD = −0.59, 95% CI: (−0.85, −0.34), *P* = 0], and the combined value of the study effect of training frequency 4–5 times per week was [SMD = −0.41, 95% CI: (−0.61, −0.21), *P* = 0.63], where training 3 times a week had a statistically significant effect on anxiety compared to the control group. For 1–2 times and 4–5 times, there were no statistically significant differences in the effects of depression between the two groups. However, the comparison between groups was statistically significant (*P* < 0.05), suggesting that the order of anxiety improvement with different training frequencies was 3 times > 4–5 times > 1–2 times.

#### Training time

Among the 10 studies included in the depression index, the combined effect of 1 study with a single training time of 50 min was [SMD = −0.24, 95% CI: (−0.49, 0.01), *P* = 0.06]. For seven single training time, the combined effect of the 60 min study was [SMD = −0.50, 95% CI: (−0.7, −0.29), *P* = 0], and the combined effect of the two studies with a single training time of 80–90 min was [SMD = −0.66, 95% CI: (−1.02, −0.29), *P* = 0]. Compared with the control group, the effect of a single training time of 50 min on anxiety was not statistically significant, and the effects of 60, 80–90 min on depression in the two groups both have a statistically significant difference. And the comparison between groups was statistically significant (*P* < 0.05), suggesting that the improvement of depression in different training time was ranked as 60 min > 80–90 min > 50 min.

Among the seven studies included in the anxiety index, the combined effect of two studies with a single training time of 50 min was [SMD = −0.27, 95% CI: (−0.48, −0.06), *P* = 0.013]. For four single training sessions, the combined value of the study effect of 60 min was [SMD = −0.53, 95% CI: (−0.79, −0.26), *P* < 0.001], and the combined effect of a single training time of 80–90 min was [SMD = −0.17, 95% CI: (−0.67, 0.34), *P* = 0.524]. Compared with the control group, the effect of a single training time of 80–90 min on anxiety was not statistically significant, and the effects of 50 and 60 min on depression in the two groups both have a statistically significant difference. And the comparison between groups was statistically significant (*P* < 0.05), suggesting that the improvement of anxiety with different training time was ranked as 80–90 min > 60 min > 50 min.

#### Precious focus

Among the 10 studies included in the depression index, the combined effect of five studies without precise focus intervention was [SMD = −0.38, 95% CI: (−0.57, −0.19, *P* = 0]. For five precise focus interventions, the combined value of the study effect was [SMD = −0.66, 95% CI: (−1.02, −0.29), *P* = 0.012]. Compared with the control group, the effect of precise focus intervention on depression was statistically significant There was a statistically significant difference between the groups (*P* < 0.05), that is, the precise focus group could significantly reduce the depression level of college students.

Among the seven studies included in the anxiety index, the combined effect of four studies without precise focus was [SMD = −0.53, 95% CI: (−0.8, −0.27), *P* = 0]. For three studies with precise focus, the combined value of the intervention study effect was [SMD = −0.66, 95% CI: (−1.02, −0.29), *P* = 0.012]. Compared with the control group, the effect of precise focus intervention on anxiety was significant statistical difference. And the comparison between groups was statistically significant (*P* < 0.05), that is, the group with precise focus could significantly reduce the anxiety level of college students.

#### Types of interventions

Among the 10 studies included in the depression index, the combined effect of 8 single taijiquan intervention studies was [SMD = −0.39, 95% CI: 95% CI: (−0.55, −0.23), *P* = 0]. The combined effect of taijiquan combined with mindfulness intervention research was [SMD = −0.52, 95% CI: (−0.75, −0.29, *P* = 0]. Compared with the control group, the effects of both intervention types on depression had a significant statistical difference. There is a statistical difference between the groups (*P* < 0.05), that is, the subgroup of taijiquan combined with mindfulness intervention can significantly reduce the depression level of college students.

Among the seven studies included in the anxiety index, the combined effect of five single taijiquan intervention studies was [SMD = −0.26, 95% CI: (−0.43, −0.09), *P* = 0]. For two studies with taijiquan combined with mindfulness, the combined effect of the intervention study was [SMD = −0.91, 95% CI: (−1.33, −0.49), *P* = 0.001]. Compared with the control group, the two intervention types had a greater effect on depression compared with the control group. There were significant statistical differences in the effects, and there was a statistical difference between the groups (*P* < 0.05), that is, the subgroup of taijiquan combined with mindfulness intervention could significantly reduce the anxiety level of college students.

### Sensitivity analysis

**Figure 5:**
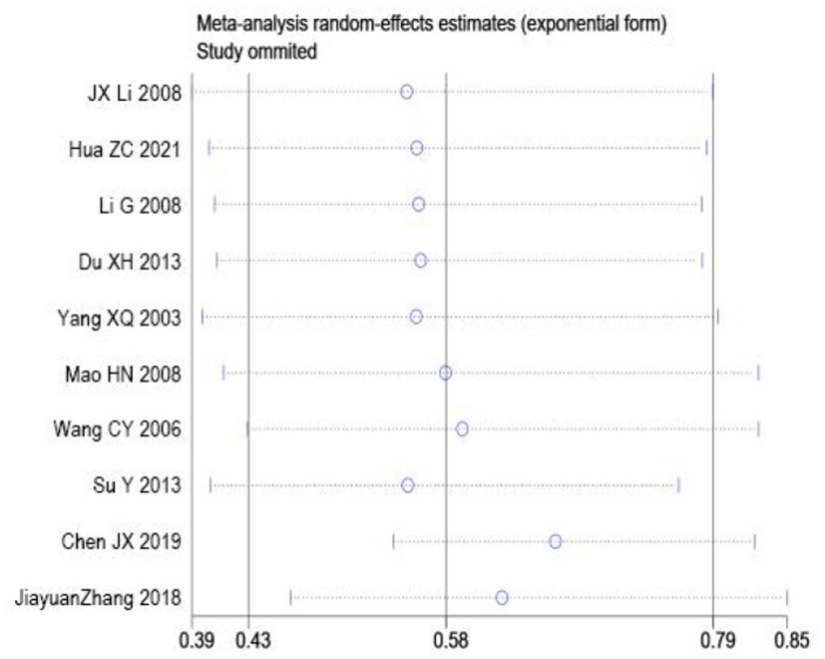
Sensitivity analysis of depression indicators.

**Figure 6:**
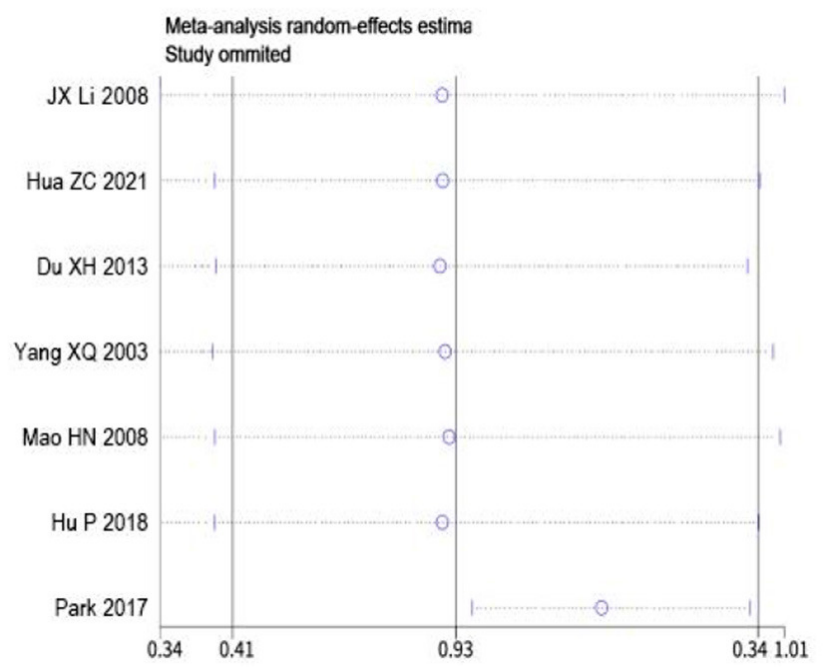
Sensitivity analysis of anxiety indicators.

Sensitivity analysis was performed on the two outcome indicators of depression and anxiety by Stata 15.0 using item-by-item elimination method. The results showed that depression and anxiety after excluding each study in turn were no more than 15% points higher than before the exclusion, and there was no significant change, indicating that the results were robust and reliable, as shown in Figures 5, 6.

### Publication bias analysis

**Figure 7:**
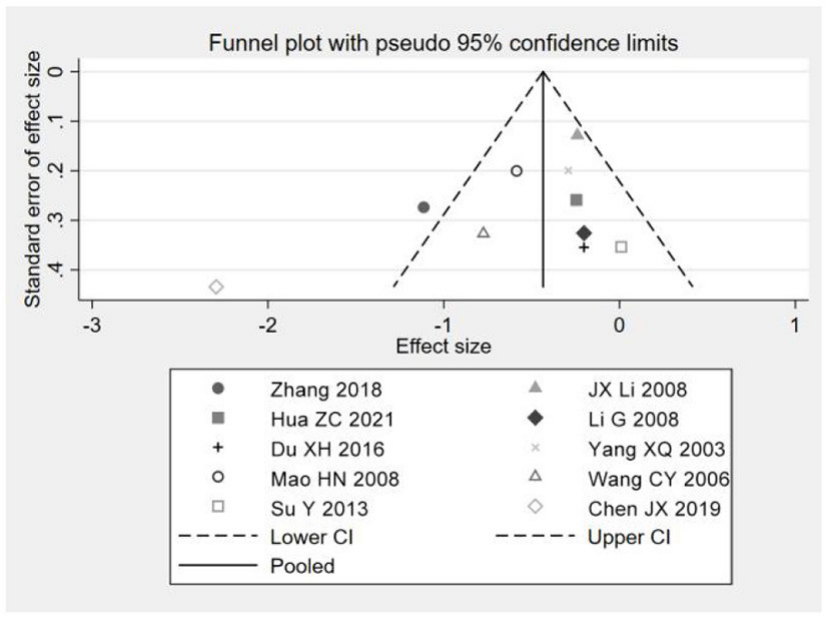
Depression index funnel plot.

**Figure 8:**
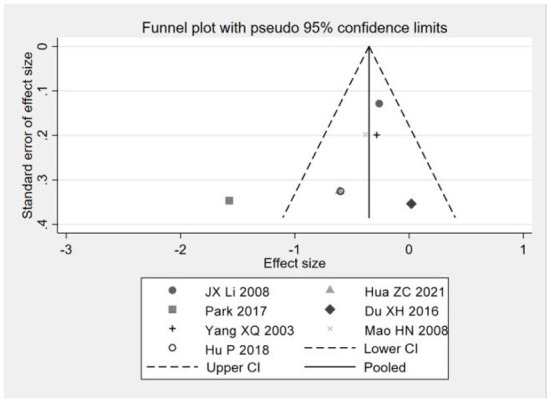
Anxiety index funnel chart.

A funnel plot combined with Egger's test and Begg's test was used to evaluate publication bias. The funnel plot results show that the distribution of studies is uneven and symmetrical, indicating that the included studies may be published, as shown in Figures 7, 8; Egger test for depression (*P* = 0.107), Begg test (*P* = 0.189) and Egger test for anxiety (*P* = 0.197), Begg test (*P* = 0.334), since *P* > 0.05 indicates that there is no obvious publication bias. Otherwise, there is publication bias. The possibility of publication bias is small. The results showed that there was no publication bias for depression and anxiety, as shown in Figures 7, 8.

## Discussion

### The influence of taijiquan on negative psychology

In interviews with clinical psychologists, foreign scholars found that because of the lack of explanatory power of exercise on the changes of clinical symptoms, clinical psychologists were willing to use exercise as a way of life rather than as a therapy (31). This paper further elaborates the mechanism of taijiquan to provide evidence for the promotion of taijiquan. Taijiquan practitioners regard “quiet mind and body relaxation” as the first essential point of practicing taijiquan (32) which is also the basic value reference and the basis of all operations of taijiquan (33). “Peace of mind” refers to the practitioner's concentration on boxing leading with mind and prompting the brain to be in a state of “self-forgetfulness” and “entry” (34). According to the physiological theory, the song-jing reaction belongs to the parasympathetic nerve in the autonomic nerve of the human body. “taijiquan's intention and effortlessness” is widely considered to be the best way to stimulate the parasympathetic nerves (35). When the human body is in a state of relaxation of the mind and body, and the parasympathetic nerves are excited, the heart rate will slow down, peripheral blood vessels will relax, blood pressure will slow down and endocrine is regulated. “Body relaxation” refers to the state where the body is as relaxed as possible during practice. Some studies have found that the influence of the EMG value of the skin surface in the waves of taijiquan relaxation exercises is regular (36). It has the function of enhancing autonomic regulation, weakening sympathetic nerves and strengthening parasympathetic nerves (37). Besides, it enhances vagal nerve regulation to improve negative psychological symptoms (37, 38). By strengthening self-control of body posture through taijiquan, it is found that in the upper center, the afferent and efferent nerve impulses are self-controlled between nerve and muscle-motor effectors at high frequency with the central nervous and peripheral sensory functions promoted and facilitated. It finally achieves therapeutic effects on the body and mind (39). Other studies have shown that taijiquan practice can also improve the practitioner's stress function (40, 41) and promote the body to secrete norepinephrine and dopamine substances (6, 42). These substances will help the body to generate exercise drive, so that the central nervous system in the body achieves a relative balance. In addition, some scholars have found that taijiquan can exert anti-depressant effects through anti-inflammatory effects during the intervention process. KIM has shown that dysregulation of inflammatory immune response is associated with the pathophysiology of depression and is one of the targets for the prevention and treatment of depression (43). Chen et al. showed that 16-week taijiquan exercise reduced pro-inflammatory factors, serum TNF-α and IL-6 levels, increased the level of anti-inflammatory factor IL-10, and decreased the level of depression for female college students (22).

### Comparison of this study with other studies

The results of this analysis further support Yang et al.'s META analysis which declares that the optimal intervention dose of physical and mental exercise for depressive symptoms is as following: more than 12-week intervention period, 3 times a week, and a single session of 60 min (44). This study further refined to obtain the optimal single intervention time for the treatment of depressive symptoms and analyzed the optimal intervention dose for the treatment of anxiety symptoms. However, different from the latter, the sing session is 80–90 min. The research of Zhao et al. suggests that a certain amount of physical exercise can effectively improve the mental health of graduate students, but the specific effects of each factor are different. This further explains the difference in the optimal single intervention time of the two indicators (45).

### Suggestions for taijiquan practitioners

As a physical and mental exercise, taijiquan emphasizes the coordination and cooperation of the three aspects of “shape, qi, and spirit” during the practice process. The research found that quite a few taijiquan therapy intervention literature only mentioned the term taijiquan intervention without indicating the focus of the intervention. Besides, they also only mentioned the term “taijiquan” without paying much attention to taijiquan's emphasis and essence of its own but only practice its moves. In this way, the effect of intervention is bound to be reduced. The subgroup analysis in this paper shows that precise focus studies can significantly improve the depression and anxiety disorders of college students (*P* < 0.05). Kim, Du et al., and other scholars believe that taijiquan therapy has no significant effect on anxiety (46, 47), which is inconsistent with the results of this study. After reviewing their research, it is found that they did not describe the intervention process too much. Therefore, the results may be different. The essentials of taijiquan were not paid attention to during the intervention, resulting in poor intervention effect. It is suggested that when conducting taijiquan intervention research in the future, emphasis should be put on “adjusting shape, breath, and mind” during the intervention process, and not just focusing on its “shape”.

### Limitations and suggestions for future research

This study was conducted in strict accordance with the PRISMA statement, but still has some limitations: There are certain differences in intervention intensity, time, and frequency among the studies, resulting in large heterogeneity. However, after sensitivity analysis and subgroup analysis, the heterogeneity of some outcome indicators has not been eliminated. Some studies have small sample sizes, which may have a certain impact on the results. Some of the included studies only focus on female college students, which may affect the reliability of the results. Future research is recommended to expand in the following aspects: First, Liu et al., Mulcahy et al., and other scholars found that there is a certain gender difference in the intervention effect of taijiquan in improving negative emotions (48, 49). Questions such as the extent to which it affects the intervention effect of taijiquan needs to be further explored. However, due to the lack of relevant literature and the limited research included in the analysis, more high-quality research needs to be carried out to verify it. Second, when conducting research on physical and mental exercise interventions such as taijiquan, the intervention process emphasizes “adjustment of shape, breath, and mind”, not just the practice of “shape”, optimizing the intervention plan, and implementing it accurately; Third, some scholars believe that a single intervention is not enough to treat anxiety disorders (50, 51). Subgroup analysis shows that taijiquan combined with mindfulness intervention can significantly reduce the negative psychological symptoms of depression and anxiety in college students than single taijiquan intervention. Mindfulness training is one of the commonly used psychological rehabilitation methods. It is based on the second generation of cognitive behavioral therapy and combined with meditation. It does not require the individual to control the internal state, and emphasizes the non-judgmental attention to the current situation and internal state to improve mental state and promote mental health (52). Many of the principles, training methods, and practice forms of taijiquan are similar to the core mechanism and training methods of mindfulness therapy. In recent years, Chinese scholars have put mindfulness training in psychotherapy. Combining it with taijiquan, mindfulness taijiquanwas created (53), and it is suggested that future research should further verify the efficacy of the combined intervention of mindfulness and taijiquan and provide evidence support for its promotion.

### Brief summary

This review analyzes the effects of taijiquan on college students' negative psychology, including depression and anxiety. The main results showed that taijiquan had a positive effect on depression and anxiety. The previous studies do not support the positive effects of taijiquan on the negative psychology of college students (37). Taijiquan therapy can significantly improve the depression and anxiety disorders of college students, with statistical significance (*P* < 0.05). According to subgroup analysis (see Table 4), the intervention period is more than 12 weeks with practicing 3 times a week, a single practice of 60 min. The intervention effect of precise focus on depression is the best. For anxiety, a single practice time comes to 80–90 min.

**Table 4:** Analysis of moderating variables.

**Outcomes**	**Moderator**	**Subgroup**	**Number of study**	**heterogeneity test**		**Sample size**	**SMD (95% CI)**	**Significance test**		**df**
				***I* ^2^**	***P***			***Z***	***P***	
Depression	Intervention cycle	≤ 12	7	44.3%	0.096	426	−0.41 (−0.62, −0.21)	−3.895***	0	6
		>12	3	90.6%	0	364	−0.45 (−0.66, −0.25)	−4.333***	0	2
	Precious focus	None	5	48%	0.1	316	−0.38 (−0.57, −0.19)	−3.961***	0	4
		Have	5	83%	0	474	−0.52 (−0.75, −0.29)	−4.361***	0	4
	Weekly practice frequency	1–2 times	3	69.7%	0.037	174	−0.55 (−0.88, −0.23)	−3.347**	0.001	2
		3 times	5	79.4%	0.001	321	−0.58 (−0.81, −0.34)	−5.023***	0	4
		4–5 times	NR	NR	NR	NR	NR	NR	NR	NR
	Single practice time	50 min	1	NR	NR	245	−0.24 (−0.49, 0.01)	−1.870	0.06	0
		60 min	7	73.5%	0.001	403	−0.5 (−0.7, −0.29)	−4.719***	0	6
		80–90 min	2	81.2%	0.021	142	−0.66 (−1.02, −0.29)	−3.486***	0	1
	Type of intervention	Single taijiquan	8	72.1%	0.001	648	−0.39 (−0.55, −0.23)	−4.834***	0	7
		Taijiquan + mindfulness	2	81.2%	0.021	142	−0.66 (−1.02, −0.29)	−3.486***	0	1
Anxiety	Intervention cycle	≤ 12	4	0	0.75	264	−0.48 (−0.75, −0.22)	−1.361	0.174	2
		>12	3	0	0.769	416	−0.28 (−0.47, −0.08)	−2.785**	0.005	2
	Precious focus of intervention	Have	4	90%	0	273	−0.53 (−0.8, −0.27)	−3.949***	0	3
		None	3	0	0.919	407	−0.25 (−0.45, −0.06)	−2.525*	0.012	2
	Weekly practice frequency	1–2 times	2	0	0.674	112	−0.1 (−0.51, −0.31)	−0.482	0.63	1
		3 times	3	93.2%	0	256	−0.59 (−0.85, −0.34)	−4.507***	0	2
		4–5 times	1	NR	NR	60	−0.41 (−0.61, −0.21)	−0.602	0.55	0
	Single practice time	50 min	2	0	0.93	347	−0.27 (−0.48, −0.06)	−2.49*	0.013	1
		60 min	4	90%	0	246	−0.53 (−0.79, −0.26)	−3.93***	0	3
		80–90 min	1	NR	NR	80	−0.17 (−0.67, 0.34)	−0.637	0.524	0
	Type of intervention	taijiquan	5	0	0.879	543	−0.26 (−0.43, −0.09)	−3.019*	0.003	4
		Taijiquan + mindfulness	2	96.2	0	130	−0.91 (−1.33, −0.49)	−4.231***	0	1

NR, not mentioned in the text.

*P < 0.05, ^**^P < 0.01, ^***^P < 0.001.

## Conclusion

The study draws the following conclusions: (1) Taijiquan therapy can significantly improve the symptoms of depression and anxiety among college students. It can be considered as a supplementary non-drug resource for depression and anxiety and has great promotion value in colleges and universities. (2) There is a precise focus on depression and anxiety symptoms. The intervention period is more than 12 weeks, and the best effect is to practice 3 times a week. The best single intervention time for depression symptoms is 80–90 min, and anxiety symptoms is 60 min; (3) The study finds that taijiquan combined with mindfulness intervention can significantly reduce the negative psychological symptoms of depression and anxiety in college students than single taijiquan intervention.

## Data availability statement

The raw data supporting the conclusions of this article will be made available by the authors, without undue reservation.

## Author contributions

All authors participated in the documentation, development, and writing of the manuscript. This study has been reviewed by all authors who are responsible for the content and final version.
